# Community engagement strategies to promote recruitment and participation in clinical research among rural communities: A narrative review

**DOI:** 10.1017/cts.2023.16

**Published:** 2023-02-08

**Authors:** Tabetha A. Brockman, Olivia Shaw, Liana Wiepert, Quang Anh Nguyen, Sydney S. Kelpin, Ian West, Monica Albertie, Shantel Williams, Adeline Abbenyi, Noreen Stephenson, Diana Almader-Douglas, Christi A. Patten

**Affiliations:** 1 Center for Clinical and Translational Science, Mayo Clinic, Rochester, MN, USA; 2 Department of Psychology and Psychiatry, Mayo Clinic, Rochester, MN, USA; 3 Virginia Polytechnic Institute and State University, Blacksburg, VA, USA; 4 Tufts University, Medford, MA, USA; 5 Luther College, Decorah, IA, USA; 6 Columbia University, New York, NY, USA; 7 Center for Health Equity and Community Engagement Research, Mayo Clinic, Jacksonville, FL, USA; 8 Education Administration, Mayo Clinic, Rochester, MN, USA

**Keywords:** Rural communities, clinical research, community engagement

## Abstract

Residents of rural areas are underrepresented in research. The aim of this narrative review was to explore studies describing the effectiveness of community engagement strategies with rural communities to promote participant recruitment and participation in clinical research. Following PRISMA guidelines, this narrative review was conducted in June 2020. Our search strategy was built around keywords that included community-engaged research, rural community, and recruitment strategies into clinical research. Content-related descriptive statistics were summarized. The selected articles were distributed into categories of levels of community engagement: inform, consult, involve, collaborate, or co-lead. The search resulted in 2,473 identified studies of which forty-eight met inclusion criteria. Of these, 47.1% were randomized controlled trials. The most common levels of engagement were consultation (n = 24 studies) and collaboration (n = 15), while very few focused on informing (n = 2) and co-leadership (n = 2). Strategies, limitations, and findings are discussed for each level of community engagement. This narrative addressed a gap in knowledge regarding participant recruitment in rural communities in relation to assistance from community members. Community engagement contributed to the success of the research, especially in recruitment, participation, and building trust and partnership.

## Introduction

Despite well-documented health disparities among rural residents, they remain underrepresented in clinical research [1]. Although approximately 14% of US residents live in rural areas, only 3% of the National Cancer Institute’s clinical research focused on rural populations between 2011 and 2016 [2,3]. Some factors contributing to this are that rural populations in America face several barriers to healthcare compared to urban and suburban populations, and clinical research studies are often offered as part of medical encounters [4]. Barriers to accessing healthcare in rural communities include geographical distances to medical facilities requiring extensive travel and lodging costs, lack of public transportation, stigma, and distrust of the healthcare system [5,6]. Representation of rural populations in clinical research is necessary to understand and deploy effective health care strategies that can benefit all rural individuals, increasing the generalizability and equity of research findings.

Recently, developing infrastructure and capabilities for clinical research to address health disparities among rural populations has been viewed as a national priority [7,8]. In response, collaborative efforts involving community-academic partnerships on community engagement, late-stage translation, and digital infrastructure have begun to address the inadequate digital infrastructure and barriers to health care access among rural communities [9,10]. A body of evidence showed that recruitment and participation of rural populations can be facilitated through community engagement in research [11,12]. Collaboration with community members has numerous merits including addressing prevalent health issues, strengthening researchers’ understanding of community priorities, and supporting culturally appropriate communication [13]. However, evidence for a comprehensive understanding of specific strategies for promoting participation in clinical research among residents of rural communities is unclear.

The goal of this narrative review is to evaluate the extant literature about the use of community engagement strategies to promote recruitment, enrollment, and participation in clinical research with rural communities. This review will highlight effective strategies in promoting participation and identify ineffective ones. This knowledge will progress the current understanding of the issue and outline specific strategies that can be applied in clinical research based in rural settings.

## Methods

To achieve the proposed objective, a narrative review was used to address the knowledge gap on ways to engage and promote clinical research in rural communities. This narrative review followed the PRISMA-ScR guidelines to plan, conduct, and report the results [14].

### Data Sources and Search Strategies

Online searches were conducted using a variety of different strategies with assistance from an experienced medical librarian. We searched PubMed, Embase, CINAHL, Scopus, Web of Science, and Cochrane Database of Systematic Reviews to identify English-language manuscripts about clinical research among rural communities. Searches of ClinicalTrials.gov were conducted to capture unpublished or ongoing research. A variety of keywords, some truncated, and subject headings were combined using Boolean operators to retrieve results in the databases. The searches included terms such as “community engaged research,” “CEnR,” “rural populations,” “recruitment,” “intervention,” “research subjects,” and “patient selection.” Results were restricted to human subjects and clinical research publication type.

### Study Eligibility and Outcome Measures

In this review, studies were eligible for inclusion if they included community engagement approaches, recruitment, and enrollment of rural participants to clinical research in the United States. Clinical research was defined as “*A component of medical and health research intended to produce knowledge valuable for understanding human disease, preventing and treating illness, and promoting health*” [15]. Key components included a sponsor, study question, study population, inclusion/exclusion criteria, observation and/or intervention, and outcomes. Types of research studies included were bi-directional integrative (translational) research, community-based clinical research, therapeutic interventions, prevention and health promotion, behavioral research, and health services research. Exclusion criteria were non-English articles, studies conducted outside the United States, systematic reviews, non-original research, and did not include rural populations. In some strategies, the text words and publication types “systematic reviews” and “meta-analyses” were also excluded. No publication period restrictions were applied to the database searches (Table 1).

**Table 1.** Summary of inclusion and exclusion criteria for the narrative review

Factor	Inclusion criteria	Exclusion criteria
Population	• Patient • Community • Rural or rural population • Rural health disparities • Veteran, military personnel • Farmer, farming	• No mention of a rural population
Setting	• Studies conducted in the United States • Studies conducted in the rural community	• International studies • No mention of rural setting
Community Engagement	• Recruitment strategies • Recruitment barriers • Peer outreach • Engagement • Patient engagement • Community engagement • Retention • Partnership • Collaboration • Community-academic partnerships • Primary care provider • Community-based participatory research • CEnR	• No mention of a community engagement component
Language	• Studies published in the English language	• Studies not published in the English language
Study Type	• Original research • Research • Clinical trial • Intervention	• Editorials, letters, chart review, conference, poster abstract, protocol papers, meta-analysis, and systematic reviews • Qualitative, interview, focus group

### Screening and Data Extraction

All records were screened among two-three reviewers by title and abstract, full text, and data extraction. Covidence was used for all study screening and data extraction activities. For data extraction and synthesis, three reviewers were assigned to each article, with decisions based on agreement among two-three of the reviewers. The following items were extracted from these studies: study design, study focus (recruitment, retention, or both), study population(s), study setting(s), number of rural research participants recruited, study description/aim, community engagement intervention for rural recruitment, level of community engagement, and major findings from the study.

The International Association for Public Participation’s Spectrum of Public Participation [16] broadly categorizes community engagement; thus, the levels of community engagement were adapted and defined as follows: (1) inform – communities are provided with information about research opportunities, (2) consult – feedback is solicited from communities for research procedures, (3) involve – communities informed and participate in research procedures, (4) collaborate – partnerships are formed with communities to work together on all procedures, and (5) co-lead – robust partnership with communities who hold decision-making power over all research procedures. If applicable, the number of urban participants was also extracted from the study. All data extraction, synthesis, and consensus were captured in Covidence for summarizing and comparisons.

### Synthesis of Results

Following the data extraction process, the information gathered was coded for the following: strategies that worked to recruit participants, strategies that did not work to recruit participants, and recruitment findings/outcomes. Three reviewers were involved in this process, and the data were then combined for one final consensus.

## Results

### Search Results

**Fig. 1.**
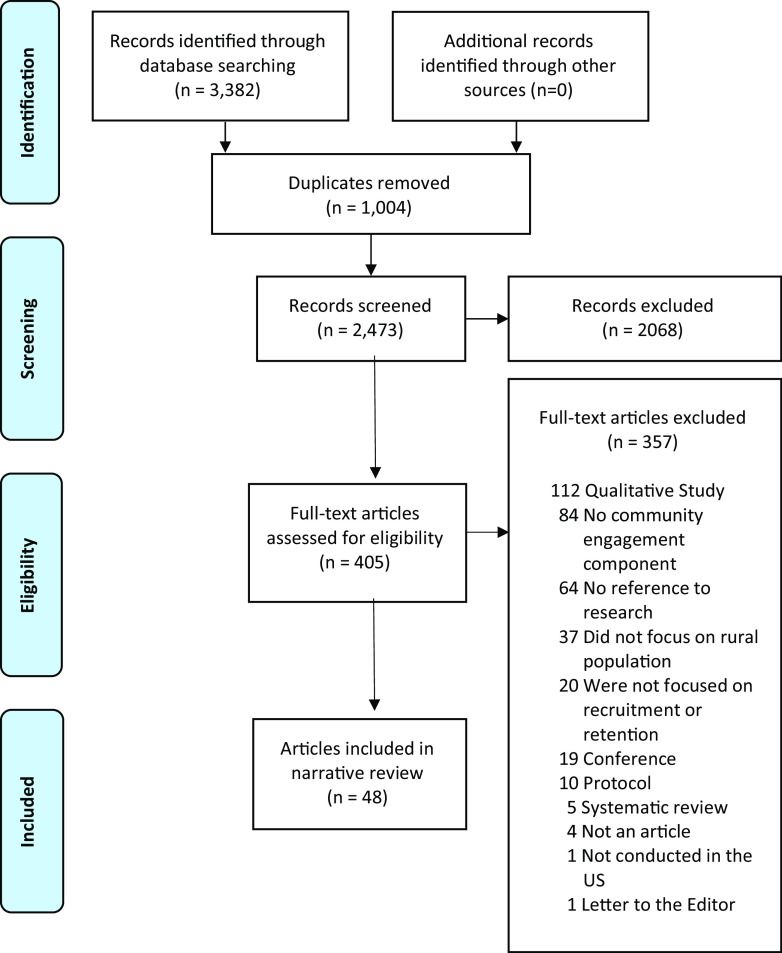
PRISMA flow diagram.

Database searches retrieved a total of 3382 papers, which yielded 2473 unique papers after de-duplication. In screening the titles and abstract, 2068 articles were excluded because of irrelevant populations, study type, setting, or missing abstract. After further screening, investigators then reviewed 405 papers of which forty-eight articles were identified to have fulfilled the inclusion criteria (see Fig. 1).

### Characteristics of Included Studies

The included studies were published between 1990 and 2021. Of the forty-eight articles, almost half (41.7%) were randomized controlled trials, and a third were quasi-experimental studies (27.1%). The remaining articles included mixed methods (8.3%), cohort designs (8.3%), cross-sectionals (8.3%), non-randomized clinical research (2.1%), case studies (2.1%), and stratified random sample studies (2.1%). The majority (91.7%) focused on rural communities, and the remaining (8.3%) included both rural and urban communities. The bulk of the included articles (70.8%) either focused on or included underrepresented groups including African American, Hispanic, American Indian or Alaska Native (AIAN) or Asian people, or the transgender community. The health-related themes of forty-eight articles were diverse; however, these were clustered into five categories: nutrient-related and nutrient processing problems (39.6%), cancer (29.2%), behavioral and physical health (27.1%), cardiovascular issues (16.7%), patient engagement (6.3%), and mental health (2.1%). Although there are some articles that only emphasized one health disparity, some had multiple. Over two-thirds (68.8%) were conducted in community settings, while 18.8% were in clinical settings and 18.8% at home or by phone. Two studies were conducted in multiple settings (Table 2).

**Table 2.** Characteristics of selected articles

First Author, Year	Study setting	Study design	# Rural & urban participants	Participant race & ethnicity	Health disparity	Level of engagement	Primary strategy(ies)	Primary outcome
Kupfer, 2009	Clinical/hospital setting	Randomized clinical trial	99 rural 416 urban	African American non-African American	Mental health	Inform	Community personnel	Improved recruitment
Gold, 1990	Home/phone	Cohort design	1168 rural	N/A	Cardiovascular issues	Inform	Community personnel Personnel remuneration	Poor outcomes Community reservation Perceived charity
Levy, 2015	Clinical/hospital setting	Quasi-experimental	351 rural	N/A	Cancer	Consult	Community personnel	Increased general health awareness
Lilly, 2014	Community setting	Quasi-experimental	81 rural	Hispanic non-Hispanic Caucasian African American	Cardiovascular issues	Consult	Community personnel Tailored materials Focus group	Improved recruitment Tailored materials
Cruz, 2014	Community setting	Randomized control trial	2444 rural	American Indian Hispanic	Nutrient-related and nutrient processing problems	Consult	Community personnel Feedback Personnel remuneration	Partnership Informed adjustment
Nguyen, 2012	Community setting	Cross-sectional survey	563 rural	African American American Indian non-Hispanic Caucasian	Nutrient-related and nutrient processing problems	Consult	Community personnel Community setting	Improved recruitment
McMahon, 2015	Community setting Home/phone	Randomized controlled trial Repeated measures	30 rural	Predominantly non-Hispanic Caucasian	Behavioral and physical health	Consult	Community personnel Feedback	Feedback Input implementation
Warren, 2010	Community setting	Quasi-experimental	188 rural	Non-Hispanic Caucasian Black Hispanic	Cancer Behavioral and physical health	Involve	Community personnel	Improved study promotion
Luque, 2017	Community setting	Quasi-experimental	90 rural	Hispanic	Cancer	Involve	Community personnel Tailored materials Community setting	Improved recruitment Tailored materials Patient-centered
Sutton, 2019	Home/phone	Cross-sectional study-community-based research	391 rural	Predominantly non-Hispanic Caucasian	Cancer	Involve	Community events Digital access Educational programs	Community personnel engagement Direct exposure to information
Yeary, 2019	Community setting	Cluster-randomized trial	426 rural	African American	Nutrient-related and nutrient processing problems	Involve	Community personnel Community setting Tailored materials	Improved recruitment Improved retention
Westfall, 2013	Home/phone	Controlled trial using a quasi-experimental repeated cross-sectional ecological design	1048 rural	Predominantly non-Hispanic Caucasian	Cancer	Involve	CBPR Tailored materials	Improved recruitment Tailored materials
Hillemeier, 2008	Clinical/hospital setting	Randomized controlled trial	362 rural	non-Hispanic Caucasian (90%) Hispanic African American Asian	Behavioral and physical health	Involve	CBPR Community personnel	Tailored materials Active recruitment Passive recruitment
Northridge, 2008	Clinical/hospital setting	Mixed methods	547 rural 155 urban	non-Hispanic Caucasian African American	Behavioral and physical health	Involve	Community personnel	Behavior change Address hard-to-reach population
Kogan, 2016	Community setting	Randomized controlled trial	465 rural	African American	Behavioral and physical health	Involve	CBPR Community personnel Tailored materials	Study adherence Intervention delivery
Dake, 2011	Community setting	Stratified random sample	778 rural	Predominantly non-Hispanic Caucasian	Behavioral and physical health	Involve	Community personnel Tailored materials	Informed adjustments Co-design
Kogan, 2016	Community setting	Observational/self-report	505 rural	African American		Involve	Community personnel	N/A
Tussing-Humphreys, 2013	Community setting	Quasi-experimental	403 rural	African American	Nutrient-related and nutrient processing problems Behavioral and physical health Cardiovascular issues	Involve	Community personnel	Educational sessions attendance
Folta, 2019	Home/phone Community setting	Randomized controlled trial	194 rural	Predominantly non-Hispanic Caucasian	Nutrient-related and nutrient processing problems Behavioral and physical health Cardiovascular issues	Involve	Community personnel Community setting Feedback	Feasible intervention delivery
Abbott, 2020	Community setting	Cluster-randomized controlled trial	146 rural	African American	Nutrient-related and nutrient processing problems Cardiovascular issues	Involve	Community personnel Community setting	Improved retention
Logan, 2015	Home/phone	Mixed method	806 rural	non-Hispanic Caucasian African American	Cancer	Involve	Community personnel Tailored materials Feedback	Feedback Tailored materials Input implementation
Hilgeman, 2014	Clinical/hospital setting	Pilot Study. prospective, randomized, controlled multi-site study Pragmatic trial	203 rural	Predominantly non-Hispanic Caucasian Black Asian Hispanic	Patient engagement	Involve	Community personnel Tailored materials	Decreased time to first appointment
Briant, 2018	Home/phone	Pre- and Post-test cohort design.	101 rural	Hispanic	Cancer	Involve	CBPR Community personnel Tailored materials	Tailored materials Improved screening rate
Jones, 2016	Community setting	Quasi-experimental	41 rural	Predominantly non-Hispanic Caucasian	Behavioral and physical health	Involve	Community personnel Tailored materials Feedback	Partnership
Befort, 2015	Clinical/hospital setting	Randomized trial	210 rural	N/A	Cancer Behavioral and physical health	Involve	Community personnel Tailored materials Community events	Improved recruitment Tailored materials Partnership
Flynn, 1997	Community setting	Randomized controlled trial-two matched sets	540 rural	Predominantly non-Hispanic Caucasian	Cancer	Involve	Community personnel Focus group Feedback	Educational program Barrier-reduction strategies
Leach, 2011	Community setting	Case study	15 rural	N/A	Cancer	Involve	CBPR Community personnel Tailored materials	Increased trust Tailored materials Improved recruitment
Estabrooks, 2017	Community setting Home/phone	Randomized controlled trial-case study	301 rural	Predominantly non-Hispanic Caucasian	Nutrient-related and nutrient processing problems	Involve	Community personnel Community setting	Improved recruitment Tailored materials
Abbott, 2018	Community setting	Cluster-randomized controlled trial	229 rural	African American	Cardiovascular issues	Involve	Community personnel Community setting	Tailored materials Behavior change
Tiwari, 2014	Community setting	CNOHR – Randomized controlled trial GIFVT – stratified randomized trial TSHS – stratified cluster-randomized trial	461 rural 597 urban	Rural American Indian Urban Hispanic, African American, and non-Hispanic Caucasian	Behavioral and physical health	Involve	CBPR Community personnel Tailored materials	Improved recruitment Increased trust Partnership Addressing community needs
Allman, 2011	Home/phone	Prospective observational cohort	366 rural 367 urban	African American, non-Hispanic Caucasian	Patient engagement	Involve	CBPR Tailored materials	Improved recruitment Increased trust
Hopper, 2017	Community setting	Community-based participatory research project. Quasi-experimental design	166 rural	Predominantly African American and American Indian Non-Hispanic Caucasian Other	Nutrient-related and nutrient processing problems	Collaborate	CBPR Community personnel	Evidence-informed intervention Community participation
Andreae, 2012	Clinical/hospital setting	Cluster-randomized trial	424 rural	African American Non-Hispanic Caucasian	Nutrient-related and nutrient processing problems	Collaborate	Community personnel Feedback	Improved recruitment Partnership
Zoellner, 2013	Community setting	Randomized controlled pilot study	91 rural	Predominantly African American	Nutrient-related and nutrient processing problems	Collaborate	CBPR Community personnel	Increased trust Partnership
Powell, 2005	Community setting	Quasi-experimental and community-based research	192 rural	African American	Cancer	Collaborate	CBPR Community personnel Tailored materials	Improved recruitment Improved retention Increased trust Tailored materials
Yeary, 2011	Community setting	Quasi-experimental	26 rural	African American	Nutrient-related and nutrient processing problems	Collaborate	CBPR Community personnel	Tailored materials Study sustainability
Valerio, 2016	Community setting	Mixed methods	117 rural	Hispanic	Patient engagement	Collaborate	Community personnel CAB	Increased trust Improved recruitment
Lemacks, 2018	Community setting	Theory-driven intervention-clinical trial	42 rural	African American	Nutrient-related and nutrient processing problems	Collaborate	Community personnel Remuneration Digital access	Improved recruitment Co-design
Vines, 2016	Community setting	Quasi-experimental	129 rural	African American	Cancer	Collaborate	Community personnel	Increased trust Partnership
Harrell, 2005	Community setting	Controlled experiment	205 rural	Racially diverse	Nutrient-related and nutrient processing problems Cardiovascular issues Behavioral and physical health	Collaborate	Community personnel Community setting Feedback	Increased participation Community support
Love, 2019	Community setting	Quasi-experimental	513 rural	American Indian	Nutrient-related and nutrient processing problems Cardiovascular issues	Collaborate	CBPR Community personnel	N/A
Davis, 2009	Community setting	Randomized controlled clinical trial	165 rural	African American Non-Hispanic Caucasian	Nutrient-related and nutrient processing problems	Collaborate	Community personnel Tailored materials	Community personnel involvement
DeMarco, 2014	Community setting	Pilot project	44 rural	N/A	Nutrient-related and nutrient processing problems	Collaborate	Community personnel Tailored materials	Increased trust
Zoellner, 2007	Community setting	Quasi-experimental	83 rural	Predominantly African American	Nutrient-related and nutrient processing problems	Collaborate	CBPR Community personnel	Tailored materials Effective delivery
Jernigan, 2018	Community setting	Cluster-randomized controlled trial	1646 rural	Native American	Nutrient-related and nutrient processing problems	Collaborate	Community personnel Tailored materials Feedback	Improved recruitment Tailored materials
Lane, 2019	Community setting	Mixed methods	13 rural	N/A	Nutrient-related and nutrient processing problems	Collaborate	Community personnel CAB	Increased participant motivation Tailored materials Behavior change
Angell, 2003	Clinical/hospital setting	Randomized controlled trial	100 rural	Predominantly non-Hispanic Caucasian	Cancer	Co-lead	CBPR Community personnel Tailored materials	Improved recruitment Improved retention Tailored materials
Schroepfer, 2009	Clinical/hospital setting	Community-based participatory research method-survey	37 rural	American Indian	Cancer	Co-lead	CBPR Community personnel Tailored materials	Improved recruitment Improved retention Tailored materials

### Recruitment Strategies

All the studies were examined in four categories: recruitment strategies, recruitment strengths, recruitment limitations, and findings (Table 3). Of the forty-eight articles included, thirty-one had a study focus on recruitment. Most of these relied on community engagement for successful recruitment of research participants. Involvement of community personnel (n = 46) [17–62], feedback (n = 22) [18,20,23,25,26,31,32,34,38,42–45,49,51–53,55–57,61,62], and customization of recruitment materials and processes in accordance with the characteristics of the community (n = 17) [19,22,25–27,32,34,45,47–50,52,56,58,61,63] were mentioned most frequently. Some other recruitment strategies included providing remuneration (either for participants or for community personnel) (n = 14) [19,23,31,33,35,37,42,44,48,49,51,54,58,61] and utilizing local spaces and events (n = 15) [19,21,22,28–30,38,39,42,46,56,58–60,63]. Although the majority of articles did not discuss limitations of the recruitment strategies, a few mentioned some challenges, including issues regarding community personnel recruitment [55,61], community personnel turnover [20,61], study interval [54], absence of educational programs [54], and ineffective recruitment via faith-based organizations [55]. Numerous outcomes of the recruitment strategies were discussed, most of which were positive. Among all the findings, production of tailored materials and procedures (n = 17) [19,25–28,31,34,45,48,51,52,56,58–60,62,64], improved participant recruitment (n = 19) [19,20,22,25–27,34,35,37,41,43,46,52,56,58,59,61–63], building partnership (n = 9) [20,23,27,40,42,49,55,56,61], and increased trust (n = 8) [23,27,35,40,50,58,61,63] were the most commonly observed outcomes. Additionally, there were other noteworthy outcomes: seven studies discussed improved participant retention [22,25,27,39,58,62,63], five studies successfully addressed community needs [52,57,61,62,64], and five studies effectively encompassed hard-to-reach populations [28,29,41,56,58].

**Table 3.** Levels of community engagement for recruitment

Level of community engagement	Definition	Recruitment approaches	Recruitment findings
Inform (n = 2)	Communities are provided with information about research opportunities.	Study team provided the communities with information via visual mediums on media platforms.	The information was successful in recruiting participants but involvement of community members in the production of these mediums would have made them more effective.
Consult (n = 5)	Feedback is solicited from communities for research procedures.	Community members provided consultation in recruitment and retention. Community members also helped validate the study to other residents.	Consultation from community personnel helped build trust and partnership between the research team and the community. Consultation also provided great insights into various aspects of research including recruitment and retention.
Involve (n = 24)	Communities are informed and participate in research procedures.	Research teams built partnerships with community organizations and local liaisons. Community members were involved directly in the process of producing materials and directly in recruitment.	Direct involvement of community members in recruitment resulted in effective recruiting, engaging hard-to-reach populations. Topics of research and information delivery methods can impact overall efficacy of recruitment.
Collaborate (n = 15)	Partnerships are formed with communities to work together on all procedures.	Community members were included in different stages and procedures, including recruitment, problem assessment, data collection, and evaluation.	Collaboration with community members at numerous stages built trust, improved recruitment, developed partnership, and produced tailored materials for research. Collaboration, if done at an early stage, can also generate well-informed intervention that is easily introduced to the community.
Co-lead (n = 2)	Robust partnership with communities who hold decision-making power over all research procedures.	Community personnel and researchers had numerous interpersonal contacts to help direct the study.	The skills and knowledge of community personnel were integrated into research. Co-leading allowed for reduction of barriers to participation and ensured that the data collected were unique and beneficial to the community.

### Levels of Rural Community Engagement

#### Inform

Among the 48 articles, only 2 involved the community at the “inform” level [41,54]. Both studies utilized visual mediums as methods of recruitment, specifically TV ads in Kupfer’s article and community presentations in Gold’s article. Although Kupfer’s study was successful in engaging African American population [41], Gold identified three shortcomings in that study including initial failure to involve community members to produce tailored educational materials, an absence of ongoing educational programs, and the short interval of the study [41].

#### Consult

There were five studies identified as engaging the community at the “consult” level [34,42,46,53,65]. The community members provided different types of support to the research teams. Although in one study, the recruited community leaders helped validate the legitimacy of the research to elder participants of the community, another demonstrated the effectiveness of a focus group which helped inform the various aspects of the intervention including acceptability, marketing, content, and environment [34,46]. The articles reported some major advantages of engaging the community members in consultation. Cruz maintained that involvement of community members who advocated for the project served to build a foundation of trust and to ensure equity in the partnership, while McMahon noted that the community partners provided advice of great value regarding maximizing participant recruitment and retention [42,53].

#### Involve

Involvement was identified as the level of engagement in half of the studies (n = 24) [17,19,21,22,26,28–30,32,33,36,38,39,45,47,48,55–61,63]. In most of these studies, the research teams developed partnerships with local organizations or recruited community personnel and liaisons. These community partners provided insights into the study processes and materials, as well as managing and executing the recruitment process. By employing local members as study staff in the recruitment, populations that would be otherwise unable to reach were engaged in the study due to the increased trust among community members, as observed in Leach’s success in involving rural Appalachian women [58]. Furthermore, the community partners also helped in tailoring materials and processes to address the communities’ specific needs and characteristics, further improving the effectiveness of recruitment. However, it must be noted that there are some limitations to involving communities to participate in the research procedures. Leach outlined that the effectiveness of community partners recruiting participants was greatly reduced due to the topic of cancer, which was too frightening, especially for those who had experienced loss of loved ones due to cancer. Moreover, some recruitment mediums in the community including emails, paid newspaper advertisements, community flyers, and postcards have also been illustrated to lack efficacy in recruiting participants [59].

#### Collaborate

Collaboration was the level of community engagement in fifteen articles [18,20,23,27,31,35,37,40,43,44,49–52,64]. In these studies, community members were involved in numerous phases of the research, including problem assessment, recruitment, data collection, and evaluation. The results of collaboration with community members were quite positive. Some of the benefits of collaboration with community personnel include increased trust, improved participant recruitment, partnership between the research group and the locals, production of specialized materials and processes, and addressing the needs of the community. Furthermore, Hopper and colleagues [18] showed that early dedication of time and resources to engage community participation generated well-informed intervention and procedures, both of which were more easily accepted and effectively disseminated within the community.

#### Co-lead

Co-leading is the driving principle regarding community personnel in only two studies [25,62]. In Angell’s study, the skills and knowledge of community personnel were integrated into the model which produced numerous interpersonal contacts between recruiters and potential participants and received great advocacy from the community [25]. In Schroepfer’s research, the community leaders who had expressed interest in the study met with university partners to assess the study’s accuracy, cultural appropriateness, and other related areas including questions involving insurance [62]. The findings from these two articles maintained that the presence of community members in every step greatly reduced barriers to participation and ensured that the data collected was distinct and bore benefits to the community, adhering to the standards of equitable and ethical research.

## Discussion

This review unveiled a body of literature regarding the use of community engagement strategies in research conducted in rural populations. The engagement of community members in research was shown to be of great benefit to study recruitment, participant participation, building partnership and trust with the community, and creation of materials and processes tailored to the community’s specific characteristics. Furthermore, engagement of community personnel in the research team ensured that the research was relevant to the community, addressed its specific needs, and benefited the community with its results. This idea of involving community members in the research team has recently been highlighted by existing health programs. A survey conducted in 2011 of the Clinical and Translation Science Awards programs demonstrated that 89% of the projects engaged Community Advisory Boards [66]. Thus, it can be maintained that the role of community engagement in research is crucial and is becoming increasingly better acknowledged within the scientific community.

The majority of studies utilized community partners to inform the research regarding recruitment, study materials, and dissemination of the intervention. Different levels of engagement imply different impacts by the community members on the research. Involvement of community personnel is most effective in the recruitment stage in which either the members provide advice on appropriate strategies, directly recruit participants, or serve as a connection between research members and residents of the community [45,47]. The presence of community members in this stage has been shown to greatly increase participant recruitment, as well as retention in some studies [63]. Moreover, involvement of community members in the research team can also foster increased trust between the researchers and the community participants [58], which is critical to facilitating effective intervention and access to hard-to-reach populations [67].

Although it was not included in the original inclusion criteria, partnership with local organizations, which is essential in establishing relationships in rural communities, was mentioned in 29 studies [17,18,22,24,26–29,32,35–43,47,48,50–52,55,56,59–61,64]. The majority of partnerships were formed with faith-based organizations (70%) and/or local health-related facilities (55%). Additionally, some other organizations involved as partners in the research were local businesses, schools or educational institutions, and general local community organizations. The importance of engaging with rural community-based organizations as a method of promoting research participation has been noted in various literature [68–70], as well as the CDC’s *Principles of Community Engagement* [71]. Culturally appropriate designs and trust in the research are important factors in engaging with the community [72], which can be facilitated through partnering with community-based organizations and receiving feedback from them. Through building solid relationships with existing local organizations, researchers can familiarize the research to a body of residents and help spread information about the project within the community. Because the majority of the selected papers did not explore the theme of participation engagement exclusively, more research exploring the effects of partnership with community-based organizations is warranted.

Successful recruitment observed in numerous studies was mostly attributed to the engagement of community members who advocated for the study. Most of the studies employed involvement and collaboration as the strategies to engage the community. Compared to information and consultation, collaboration, and involvement transfer some of the decision-making ability to the community members, as they can directly make decisions that impact the studies, evoking a sense of ownership and responsibility toward the research. In a corporate setting, involving employees in the decision-making process can induce a sense of ownership, produce an alignment of interest, and improve overall productivity and quality [73]. In the setting of clinical research, this can translate into improvement of the quality and effectiveness as observed in the selected articles. Additionally, with more power, community members can positively influence the procedures, strategies, and materials to ensure they best address and benefit the members of the community.

Of the forty-eight studies, only two employed co-leadership as the method of community engagement; however, the findings were very promising. In Angell’s article, efforts made by the community and their input were depicted as indispensable components to the research’s success. Moreover, Schroepfer’s study demonstrated that co-leadership empowered the community, enhanced the capacity of conducting research, and allowed for interpretation of data within the framework of local knowledge, available resources, specific values and beliefs [25,62]. These positive results certainly highlight the need for future research employing co-leadership as the engagement principle. Notably, both community personnel recruitment and turnover were mentioned as some of the potential challenges of this approach to consider in future research.

Only seven of the studies focused on implementing community engagement strategies among American Indian and Alaska Natives (AIAN) persons [18,42,44,46,52,61,62]. This is important to consider as AIAN communities remain disproportionately rural compared to other groups, with 29% identified as living in rural areas in 2010 compared to 15% of the total US population [74]. Comprising a large part of rural communities, AIAN people must be included in clinical research in order to learn effective community engagement strategies within this population. Moreover, community engagement techniques may be particularly beneficial when working with AIAN people due to research historically often not addressing community priorities and with little regard for cultural practices [75]. Furthermore, research has often been done without seeking consent from Indigenous communities or not communicating clearly when obtaining consent [76]. Recent initiatives have called for the need for more culturally sensitive and collaborative approaches in research with indigenous populations [77,78]. Community engagement strategies could provide a necessary framework for such efforts; however, more research is needed to better understand best practices within this population.

There are several limitations to the present review. While each level of engagement demonstrated unique benefits to the research, none of the included studies directly compared the different levels of engagement. In future research, it will be beneficial to understand the context in which each level would yield the highest contribution to the successes of the study. Furthermore, knowledge of different engagement principles will also determine balancing community involvement in decision-making and maintaining scientific vigor. Our narrative review is limited to research conducted in the United States and studies published in the English language, limiting generalizability to other countries and languages. Future narrative reviews that include international studies are needed to learn from the work of our neighbors and international partners such as Australia and Canada, who are working on similar issues. Moreover, this review highlights that compared to the whole, CEnR with rural populations occupy only a small portion of the existing body of research. Hence, more CEnR is needed in rural settings that are often underrepresented in the scientific world [4]. Additionally, specific definitions and characteristics of rurality were not captured in the present review (e.g., town size). Rurality is a multidimensional concept, and there is great variation across rural communities that need to be considered when implementing community engagement strategies.

## Conclusion

This review highlighted the sizeable contributions community members and community leaders can have to the success of research. Most of the studies used collaboration and involvement as the principle driving community engagement. It can be easily observed that including community members on the research staff can enhance the quality of the research, appropriateness of the materials, and effectiveness of intervention delivery. Although it contains many benefits, community engagement is not the omnipotent answer to tackle every barrier, such as transportation, geological isolation, or insufficient services and infrastructure, all of which contribute to the exclusion of rural populations in clinical research. However, it is a necessary component to include in any solution to resolve these issues. Community engagement strategies produce more equity in clinical research, as the participants are part of the team and can ensure that the research is appropriate and beneficial to community. Additional research on community-engaged strategies to enhance clinical research participation among rural populations is warranted [4].
